# Single cell transcriptomic representation of social dominance in prefrontal cortex and the influence of preweaning maternal and postweaning social environment

**DOI:** 10.1038/s41598-024-52200-6

**Published:** 2024-01-25

**Authors:** Katherine Lopez, Madelyn R. Baker, Miklos Toth

**Affiliations:** 1 Department of Pharmacology, Weill Cornell Medicine, 1300 York Ave, New York, NY 10065 USA; 2 Neuroscience Program, Weill Cornell Graduate School of Medical Sciences, 1300 York Ave, New York, NY 10065 USA

**Keywords:** Social behaviour, Transcriptomics

## Abstract

Social dominance encompasses winning dyadic contests and gaining priority access to resources and reproduction. Dominance is influenced by environmental factors, particularly during early postnatal life and adolescence. A disinhibitory medial prefrontal cortex (mPFC) microcircuit has been implicated in the expression of dominance in the “tube test” social competition paradigm in mice, but the neuroplasticity underlying dominance is not known. We previously reported that male pups raised by physically active (wheel-running, as opposed to sedentary) dams exhibit tube test dominance and increased reproductive fitness, and here we show that social isolation from weaning also increases dominance. By using single cell transcriptomics, we tested if increased dominance in these models is associated with a specific transcriptional profile in one or more cell-types in the mPFC. The preweaning maternal effect, but not postweaning social isolation, caused gene expression changes in pyramidal neurons. However, both the effect of maternal exercise and social isolation induced the coordinated downregulation of synaptic channel, receptor, and adhesion genes in parvalbumin positive (PV) interneurons, suggesting that development of dominance is accompanied by impaired PV interneuron-mediated inhibition of pyramidal cells. This study may help understand environmentally induced transcriptional plasticity in the PFC and its relationship to tube test dominance.

## Introduction

Dominance/subordinance is a social, dyadic behavior with one individual exhibiting or signaling aggressive behavior and the other responding with submissive behavior^1,2^. However, dominance is more than direct aggression because it includes cognitive skills, assertiveness, and vigilance to achieve and maintain high social status in a group^3–5^. Dominant individuals have preferential access to resources, shelter, and mates, and have higher reproductive fitness than subordinates^6^. Non-aggressive dominance is not limited to humans and has been observed in rodents both in the wild and in the laboratory^7–9^.

Animals living in social groups form a dominance hierarchy through repetitive social interactions both in the wild and in controlled laboratory environments^1,10^. Winners of previous competitions are more likely to keep winning future contests and thus establish a dominant status^11^. However, social dominance is a broader concept than social rank in a group because dominance is exhibited against individuals outside of the group and socially isolated individuals, with no social experience, exhibit dominance. Social dominance can be assessed by various methods^12,13^. The “tube test” emerged as a simple, reliable, and reproducible method to determine social rank within a group of rodents, typically mice^14^. In this test, two animals confront each other in a Plexiglas tube, with the “winner” pushing the “loser” out from the tube or the “loser” withdrawing from the tube^14^. Although tube test dominance may operationally be defined as the ability to monopolize priority access to prized resources, tube test measures may not be generalized across other dominance tests^15^.

Genetics plays a role in exhibiting dominance behavior, exemplified by the tube test dominance of individuals from one inbred strain over individuals from another inbred strain^9^. Nonetheless, males with the same genetic background display variable levels of dominance in tube tests against unfamiliar competitors^9^. Nongenetic factors that may influence dominance behavior include variations in the prenatal environment, such as position in the uterus, and early postnatal environment, including differences in maternal care^16–19^. We previously identified a maternal factor that reliably influences the social dominance of males in the tube test^20^. We reported that daily voluntary wheel running of dams, from postpartum day 2 to 21, increases the tube test dominance and reproductive success/fitness of their adult “Run” offspring, as compared to the sedentary mothers’ “Sed” offspring^20^.

Another nongenetic factor that was reported to increase social dominance is social isolation. Social isolation of rodents during the neonatal and juvenile (from weaning) periods has consistently been shown to result in abnormal social behavior, including hyperactivity, aggression, increased dominance, and impaired sociability^21–25^. Similar behavioral changes have also been observed in humans as a result of childhood neglect or abuse, characterized by excessive dominance, overt aggression, antisocial behavior, greater than normal reward sensitivity, and inflated self-perception^26–28^.

Tube test social rank within a group of cage mates has been reported to be bidirectionally controlled by synaptic efficacy in the medial prefrontal cortex (mPFC)^13^. Another report showed that both the winner’s and loser’s behavior in the tube test (pushing and resisting, respectively) is correlated with neuronal activity in the prelimbic (PL) area of the mPFC^29^. Finally, a recent work identified a disinhibitory microcircuit in the dorsal mPFC, that through two types of interneurons to pyramidal neurons, controls tube test competition^30^. We speculated that the increased tube test dominance of Run and socially isolated males could be associated with neuronal changes in this mPFC circuit and that dominance-related neuroplasticity in this circuit is reflected in transcriptomic alterations. Because the mPFC circuit regulating dominance behavior is composed of both pyramidal neurons and different types of interneurons, we used single nucleus (sn) RNA-Seq to determine cell-type specific gene expression profiles and the influence of the preweaning Run maternal environment and postweaning social isolation on gene expression in the mPFC. We found transcriptional changes in the PL that were specific for dominance, independent of whether attained by the preweaning Run maternal environment or social isolation, in parvalbumin (PV) positive interneurons. Transcriptional changes suggested reduced inhibition of pyramidal neurons by interneurons, a model consistent with dominance driven by the disinhibition of pyramidal neurons in the mPFC.

## Results

### Male offspring raised by postpartum exercising mothers are socially dominant relative to the offspring of sedentary mothers

**Figure 1:**
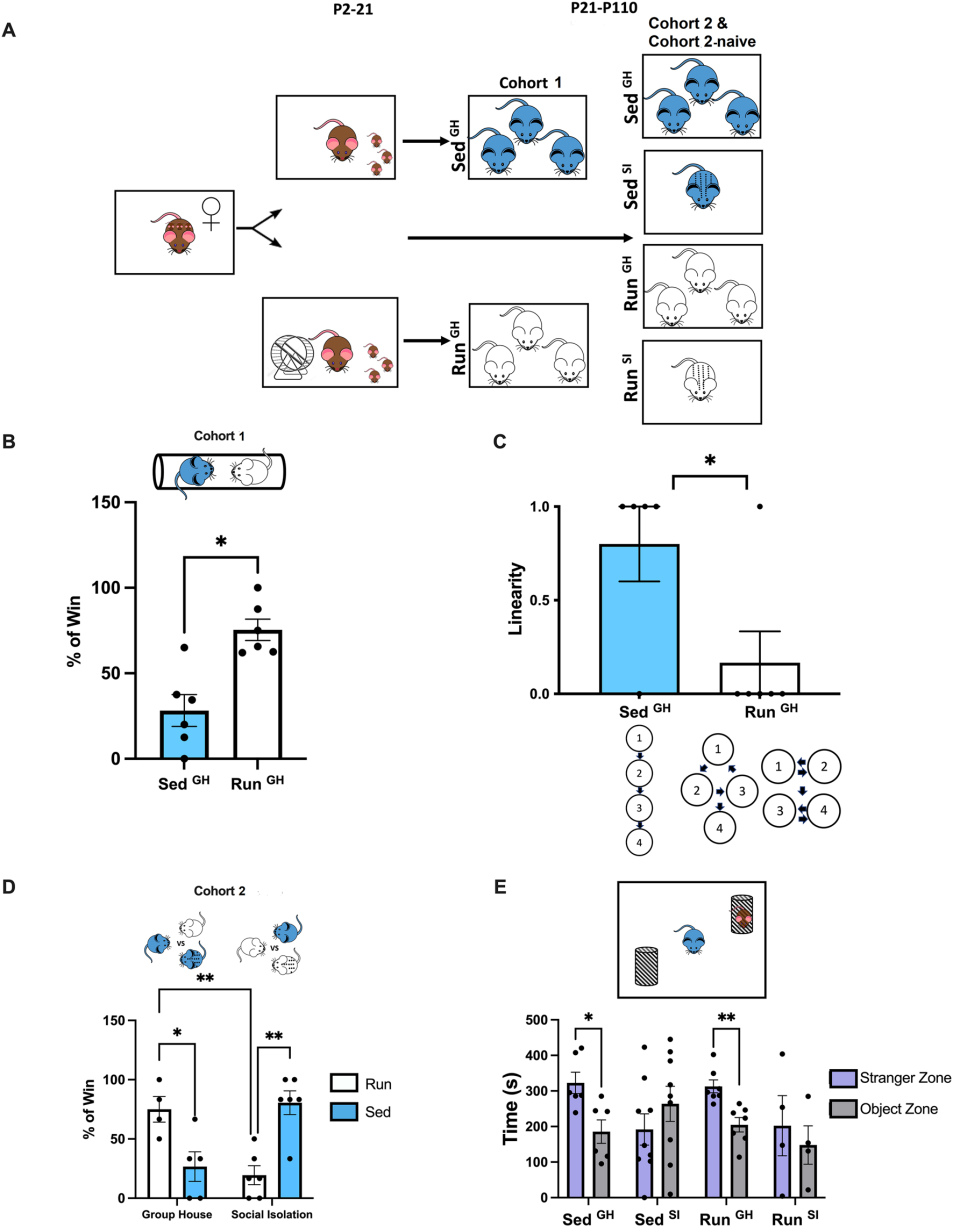
Maternal postpartum physical activity and social isolation increase social dominance of males in the tube test. (**A**) Experimental design to generate Run and Sed offspring of mothers housed in cages with or without running wheels during the postpartum period. Both Run and Sed males were divided at P21 and raised under group housing condition (Run^GH^ and Sed^GH^, cohorts 1 and 2) or in social isolation (SI) in single housing condition (Run^SI^ and Sed^SI^, cohort 2). None of the offspring had running wheel in their cage. (**B**) Adult (~ 4 months old) Run^GH^ males were dominant over Sed^GH^ males in the tube test. Unpaired t test, N = 6 (Run), 6 (Sed); t(10) = 4.205, **p = 0.0018. Data are represented as mean +—SEM. (**C**) While groups of Sed^GH^ male cagemates predominantly showed linear hierarchy, maternal running resulted in loss of linearity in the groups of Run^GH^ offspring in “within cage” tube tests, using a round robin design. Mann–Whitney test, U = 5.500, p* = 0.040. (**D**) Run^GH^ males were dominant over Sed^GH^ males, while social isolation elevated the dominance of Sed^SI^ males over Sed^GH^ males. Two-way ANOVA, Interaction between maternal (Run, Sed) and offspring (GH, SI) environments F(1,17) = 27.62, P < 0.0001. Tukey’s test for multiple comparisons: Sed^GH^ vs. Run^GH^, *p = 0.0323; Sed^SI^ vs. Sed^GH^, **p = 0.0074; Run^GH^ vs. Run^SI^, **p = 0.0095. N = 5 (Sed^GH^), 6 (Sed^SI^), 4 (Run^GH^), 6 (Run^SI^). (**E**) Group-housed males (both Run^GH^ and Sed^GH^) had increased preference for interacting with a caged stranger mouse over an empty cup, while preference was lost in socially isolated males (both Sed^SI^ and Run^SI^). Multiple paired t tests, Sidak’s multiple comparison: Sed^GH^, *p = 0.049752; Run^GH^, **p = 0.00907. Although the same cohort was used as in D, more mice were available because, unlike the tube test that used select individuals for making pairs, affiliative behavior could be assessed with all available males. However, three Run^SI^ and one Run^GH^ males were excluded because climbing to cups or attempting to jump from the enclosure. N = 6 (Sed^GH^), 9 (Sed^SI^), 7 (Run^GH^), 4 (Run^SI^).

We previously reported that the preweaning maternal environment has a strong influence on the individual’s adult social dominance in the tube test in C57BL/6 mice^20^. Specifically, male but not female pups raised by dams in cages equipped with a running wheel during postpartum days 2 to 21 (Run offspring) exhibited dominance in the tube test over unfamiliar males raised by mothers in standard cages (Sed offspring) (Fig. 1A). Exercise on a running wheel is a naturalistic behavior^31^ and mothers used it voluntarily without neglecting their pups^20^. Here we reproduced the increased dominance phenotype of group-housed (GH) Run males (Run^GH^), indicated by their significantly higher winning score against unfamiliar, age- and weight-matched group-housed Sed^GH^ male offspring of sedentary mothers (Fig. 1A cohort 1**,** Fig. 1B). Each male was from a separate mother (e.g., males were not littermates) and each pairwise competition was performed twice by switching competitors between the sides of the tube to eliminate possible side bias. Further, to limit the effect of winning history on dominance^11^, each male competed with 2–3 males from the other group. The social dominance behavior of the male offspring was fairly specific for the tube test, as maternal exercise had no effect on urine marking, another measure of social dominance, and on agonistic behavior in direct pairwise interactions with conspecifics, as we reported previously^20^.

Next, we assessed dominance hierarchy within the groups of Run^GH^ and Sed^GH^ mice in a round-robin competition between cage mates (Fig. 1C, cohort 1). While 4 out of the 5 Sed^GH^ groups formed a linear hierarchy, only 1 out of the 6 Run^GH^ groups showed linearity in rank as multiple males achieved dominance status in 5 Run^GH^ cages. Since Run males, as a group, were dominant over Sed males (Fig. 1B), we concluded that the exercise-related maternal effect elevates dominance behavior above that which is achieved within a group of Sed cage mates.

### Postweaning isolation increases dominance of Sed males but reduces the dominance of Run males

Neonatal social isolation has been reported to increase the dominance of male mice in the tube test^21^. To test if social isolation, from weaning (postnatal day, P21) to testing in adulthood (~ 4 months of age), a period that spans the juvenile and adolescent development, leads to a similar behavioral phenotype, we generated a new group of Run and Sed mice and divided them at weaning to group-housed and socially isolated (SI) groups, thus creating four groups of mice (Run^GH^, Sed^GH^, Run^SI^, and Sed^SI^) (Fig. 1A, cohort 2). In adulthood, individuals from each group competed against individuals from other groups that differed from them in one variable, either in preweaning maternal effect (Run/Sed) or postweaning social environment (GH/SI) (Fig. 1D). To avoid bias due to winning history^11^, each male competed with only 2–3 males from the other groups.

We again reproduced the dominance of Run^GH^ over Sed^GH^ males (Fig. 1D). Further, we found that socially isolated Sed males (Sed^SI^) exhibited increased dominance over group-housed Sed (Sed^GH^) males (Fig. 1D), indicating that social isolation beyond the neonatal age can still increase tube test dominance. In contrast, socially isolated Run males (Run^SI^) became submissive when competing against group-housed Run males (Run^GH^) (Fig. 1D), indicating an opposite effect of social isolation in Run and Sed mice.

To test the relationship between tube test dominance and social motivation^32^, a different social behavior, we also assessed the behavior of these, cohort 2 males in the 3 chamber social interaction test^33^ (Fig. 1E). Social isolation abolished the social preference (i.e., preference for a caged stranger mouse vs. empty cup) of both Run^SI^ and Sed^SI^ mice. Further, Run^GH^ and Sed^GH^ males had a similar social preference indicating that tube test social dominance is unrelated to social motivation to interact with a conspecific.

Overall, these data show that tube test dominance of males can be increased by two distinct environmental effects, maternal postpartum physical exercise during the preweaning period and postweaning social isolation.

### Cellular composition of the PL mPFC

**Figure 2:**
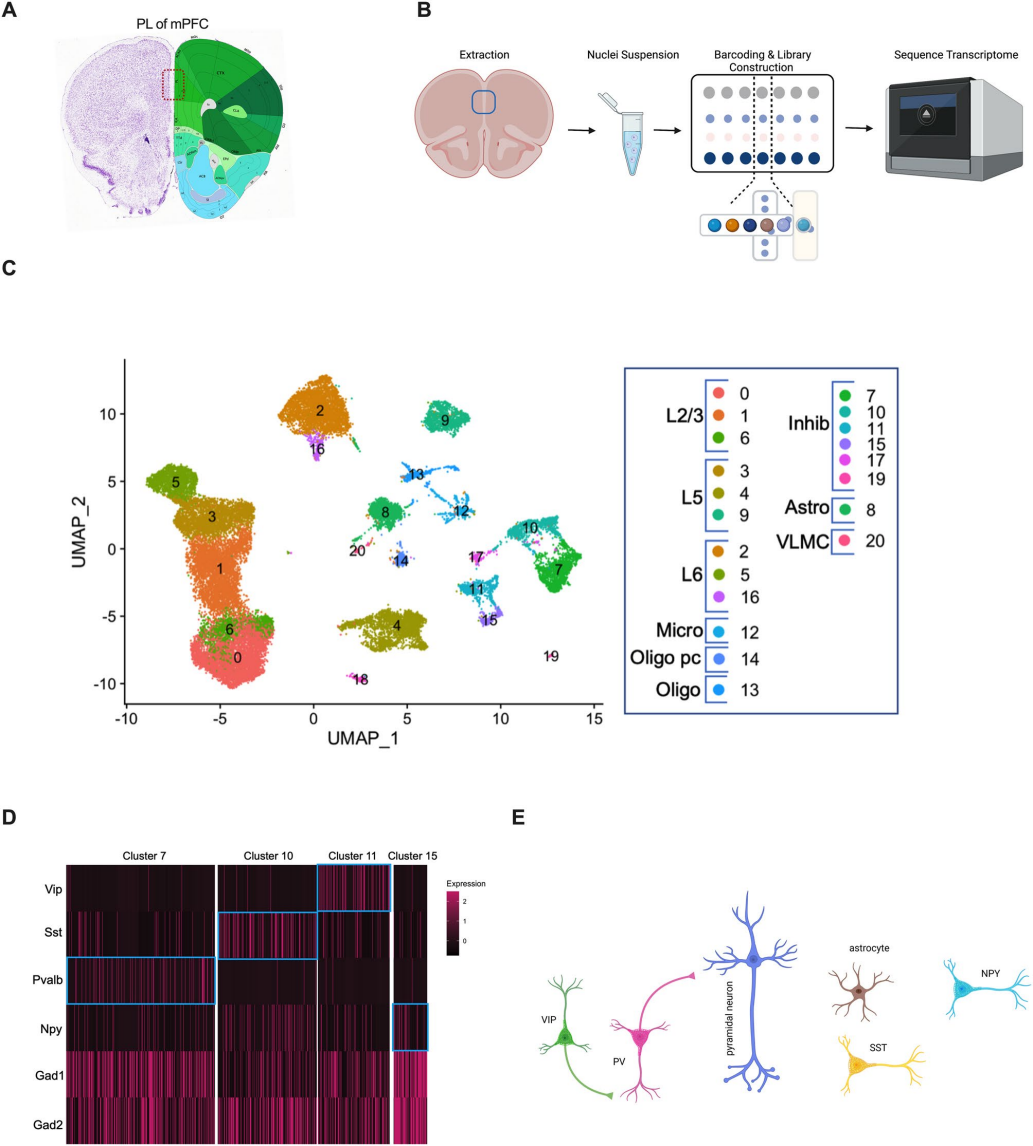
Cellular composition of the PL analyzed by snRNA-Seq on the 10X Chromium platform (10X Genomics). (**A**) PL of mPFC was isolated from coronal sections as indicated. (**B**) Isolated PL was used for nuclei isolation and snRNA-Seq. (**C**) UMAP plot of transcriptional profiles of single nuclei showing all identified neuronal and nonneuronal cell types. Data are from the four-group snRNA-Seq experiment (cohort 2-naïve, *dataset 2*). L = cortical layer, Micro = microglia, Oligo pc = oligodendrocyte precursor, Inhib = inhibitory neurons, Endo = endothelial cell, VLMC = vascular and leptomeningeal cells. (**D**) Single cell heat map showing cell type-specific gene marker expression in identifiable interneuron clusters. (**E**) The disinhibitory microcircuit, composed of pyramidal neurons, PV, and VIP neurons, implicated in the regulation of dominance behavior in the tube test. Additional cell types identified in the PL and analyzed for gene expression are also shown.

Because dominance is linked to neuronal plasticity in a disinhibitory neuronal circuit in the PL and anterior cingulate cortex (ACC) regions of the mPFC^30^, we tested if the increased tube test dominance of group-housed Run (Run^GH^) and socially isolated Sed males (Sed^SI^) is accompanied by PL transcriptional changes. Given that the PL, and the mPFC in general, are composed of several neuronal and nonneuronal cell types that might be involved in the regulation of social dominance, we used single cell-based transcriptomics to characterize the influence of the preweaning maternal and postweaning social environment on cell-type specific gene expression. Although single cell transcriptomic profiling of the mouse mPFC has been reported^34,35^, our focus on the PL, which is the middle part of the mPFC (Fig. 2A), motivated us to generate a PL-specific cell atlas. Further, we used nuclei, rather than cells, because nuclei isolation (Fig. 2B), as compared to enzymatic digestion of the tissue into single cell suspension, avoided the activation of immediate early genes^36^. Additionally, nascent nuclear transcripts may better reflect transcription rate as their level is not influenced by variability in nucleo-cytoplasmic transport and stability in the cytoplasm.

First, we performed an snRNA-Seq experiment from cohort 1 Run^GH^ and Sed^GH^ mice with PL nuclei isolated one day after the last tube test (at ~ 4 months of age), generating *dataset 1* (Fig. 1A, B). Three adult Run and Sed males that ranked top and bottom within their own group, each from a different mother, were used for dissecting PL, followed by nuclei isolation and 10 × profiling as shown in Fig. 2B. 10,232 Run^GH^ and 7,327 Sed^GH^ nuclei passed quality control and were clustered by Uniform Manifold Approximation and Projection (UMAP) using Seurat^37^. A similar snRNA-Seq experiment was performed with an independent cohort of ~ 4 months old mice that followed the 4-group experimental design used for behavioral cohort 2, and which showed the dominance behavior of both Run^GH^ and Sed^SI^ offspring in competition with Sed^GH^ mice (Fig. 1A, D). This cohort (cohort 2-naïve) however had no prior exposure to the tube or other behavioral tests (e.g., *dataset 2*). PL from 3 Run^GH^, 3 Sed^GH^, 3 Run^SI^, and 3 Sed^SI^ mice (each from a different mother) yielded 10,272, 4,939, 9,159, and 5,360 nuclei, respectively, that passed quality control. The number and size of UMAP clusters were similar with the Run^GH^ and Sed^GH^ groups across *datasets 1* and* 2*, although the position of clusters within UMAPs varied slightly in the two experiments (Supplementary Figure S1A, B). Further, the UMAP patterns were indistinguishable across groups within *dataset 2* indicating no apparent effect of the preweaning or postweaning environment (Supplementary Figure S1B). Finally, total gene expression levels between the two sets of Run^GH^ and Sed^GH^ PL were highly correlated (Pearson’s correlation, r = 0.96) indicating that prior behavioral testing had no obvious effect on overall gene expression in the PL (Supplementary Figure S1C).

We used the combined UMAP profile of the four groups with no prior behavioral testing (*dataset 2*) to assign clusters to cell types, based on cell type specific markers reported earlier in the mouse mPFC^35^ (Fig. 2C and Supplementary Figure S2 for cluster markers). We identified 9 clusters corresponding to excitatory pyramidal neurons in the PL (Fig. 2C). These excitatory neuronal clusters could be assigned to specific cortical layers using layer-specific cell type markers (Supplementary Figure S2). Clusters 0, 1, and 6 corresponded to layer 2/3 pyramidal neurons (based on their expression of *Cux2*), clusters 3, 4, and 9 corresponded to layer 5 neurons (*Etv1* +), with cluster 9 corresponding to a *Tshz2* + subtype of layer 5 neurons, and clusters 2, 5, and 16 corresponded to layer 6 neurons (*Syt6* + , *Foxp2* + , *Oprk1* +). Due to the smaller number of interneurons (*Gad1* + *, Gad2* +), their subtype classification is more challenging. Nonetheless, we identified all four major subtypes of interneurons, PV (cluster 7, *Pvalb* +), somatostatin (SST, cluster 10, *Sst* +), vasoactive intestinal peptide (VIP, cluster 11, *Vip* +), and neuropeptide Y (NPY, cluster 15, *Npy* +)^38^ (Fig. 2D, Supplementary Figure S2, Supplementary Table S1, dataset 2). In addition to neuronal subtypes, we identified non-neuronal cells such as astrocytes (cluster 8, *Gja1*^+^). Additional clusters corresponded to oligodendrocytes (cluster 13, *Mbp*^+^), oligodendrocyte precursors (cluster 14, *Pdgfra*^+^), and microglia (cluster 12, *Cx3cr1*^+^) (Fig. 2C). Given the small number of nuclei corresponding to oligodendrocytes and microglia, they were not analyzed further. Of note, the same cell types and cell type specific clusters were also identified in the UMAPs generated by *dataset 1* (Supplementary Figure S1A and Supplementary Table S1, dataset 1).

Overall, we show that individual neuronal and non-neuronal cell types can be identified by snRNA-Seq from the relatively small region of the PL of the mPFC. Some of these cell types, specifically pyramidal neurons and PV and VIP interneurons, have been implicated in forming a disinhibitory mPFC microcircuit to regulate social competition in the tube test^13^ (Fig. 2E).

### The preweaning run maternal environment induces gene expression changes in a wide range of cell types in the PL

Next, we used the two-group (Run^GH^ and Sed^GH^ with prior behavioral testing, cohort 1) and four-group (Run^GH^, Sed^GH^, Run^SI^, and Sed^SI^, no prior testing, cohort 2-naive) snRNA-Seq datasets (*datasets 1* and *2*, respectively) for differential expression analysis with Seurat^37^ to determine whether dominance behavior is associated with gene expression changes in one or more cell types in the PL.

Analysis of the two-group (Run^GH^ and Sed^GH^) dataset (*dataset 1*) showed 35, 30, and 56 differentially expressed genes (DEGs, ≥ 1.3-fold change, adjusted *p* value ≤ 0.05) between the two groups in layer 2/3, 5, and 6 pyramidal neurons, respectively, exhibiting around 50% gene overlap (Supplementary Figure S3A, Supplementary Table S2). This suggested a shared transcriptional response of pyramidal neurons to the preweaning Run maternal environment. Because of the overlap between layer specific DEGs and their modest numbers, we combined all pyramidal neurons that yielded a set of 60 PL pyramidal neuron DEGs (25 up- and 35 downregulated (Supplementary Figure S3B). This set of DEGs showed enrichment in biological processes related to synapse and neurite development and function (Ingenuity Pathway analysis (IPA), Benjamini-Hochberg (B-H) corrected p-values from 3E-6 to 3E-4) (Supplementary Figure S3C). Of note, a layer-specific analysis produced similar results with higher, but still significant (< 0.05) B-H *p*-values.

In PV interneurons, a similar number of Run^GH^ versus Sed^GH^ DEGs were found, but they were mostly downregulated (20 up- and 42 downregulated, Supplementary Table S2 and Supplementary Fig. 4A) and were enriched at a higher level in IPA biological processes (up to 2E-7) (Supplementary Fig. 4B). The identified biological processes suggested the involvement of Run^GH^ vs. Sed^GH^ DEGs in the neuronal, neurite, and synapse development of PV interneurons and many of them were associated with a negative z score. These data are consistent with impaired PV function reported in high-ranking dominant male mice^30^. SST and VIP interneurons and astrocytes had too few DEGs (26, 34, and 25, respectively) for a functional analysis and were not analyzed further. These data indicate that the preweaning maternal environment resulted in significant transcriptional changes in pyramidal neurons and PV interneurons.

**Figure 3:**
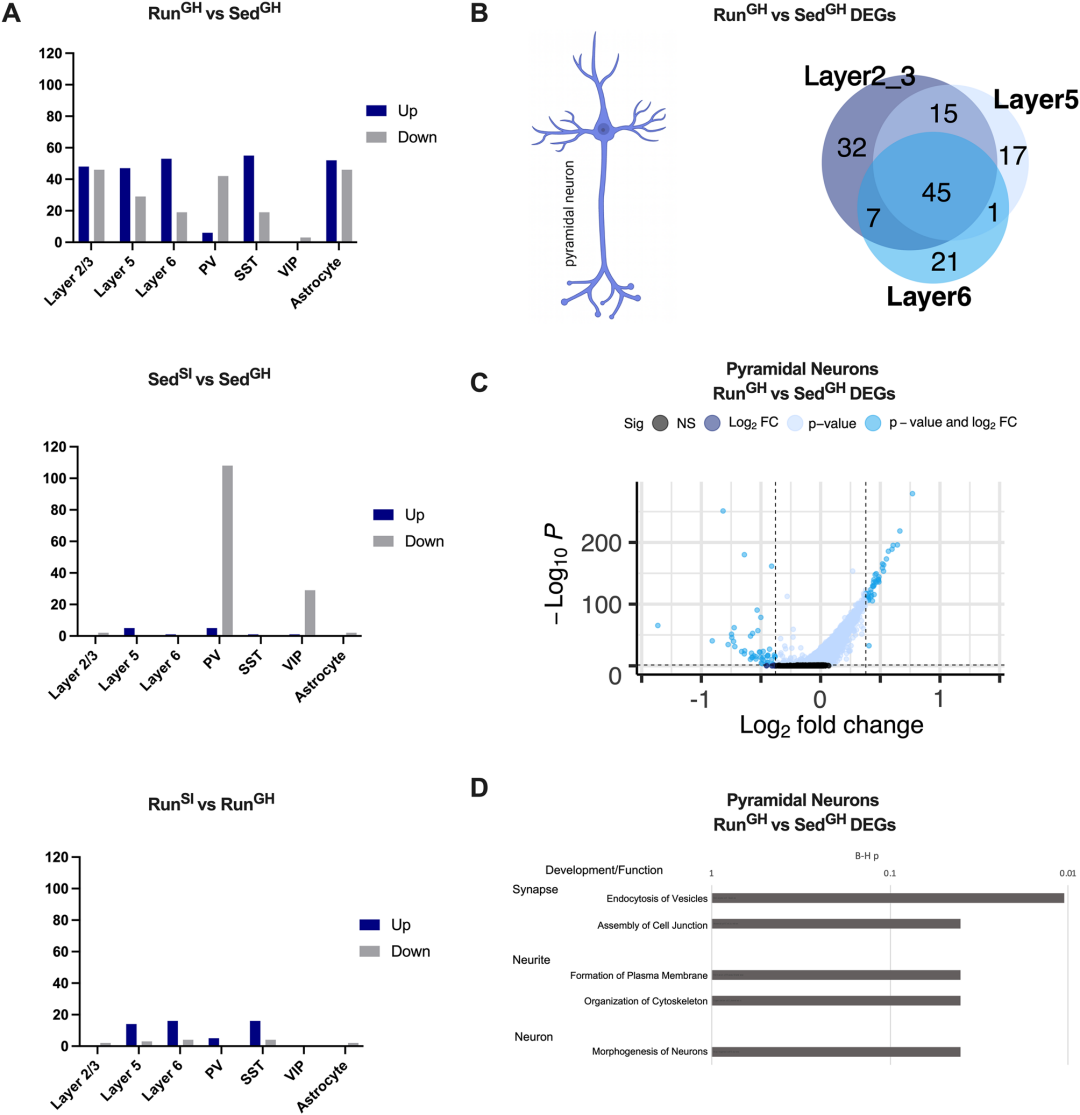
Differential gene expression in various PL cells as a function of the preweaning maternal environment and postweaning social isolation. Data are from the four-group snRNA-Seq experiment (cohort 2-naïve, *dataset 2*). (**A**) The preweaning maternal environment results in differential expression in multiple PL cell types (Run^GH^ vs. Sed^GH^, top), while postweaning social isolation affects gene expression predominantly in PV interneurons (Sed^SI^ vs. Sed^GH^, middle). Social isolation of Run males results in minimal gene expression changes across PL cell types (Run^SI^ vs. Run^GH^, bottom). (**B**) Pyramidal neuron specific Run^GH^ vs. Sed^GH^ DEGs in the three layers of PL highly overlap. (**C**) Volcano plot representation of gene expression differences caused by the maternal Run environment in pyramidal neurons in the PL. (**D**) IPA biological processes enriched in Run^GH^ vs. Sed^GH^ pyramidal neuron DEGs.

Next, we used the four-group snRNA-Seq dataset (*dataset 2*, Run^GH^, Sed^GH^, Run^SI^, and Sed^SI^, no prior behavioral testing, cohort 2-naive) that allowed us to study the transcriptomic effects of both the preweaning maternal and postweaning social isolation in the different mPFC cell types (see UMAP distribution of cell types of this group of mice in Fig. 2C and Supplementary Figure S1B). First, we verified that, like with the previous groups of Run^GH^ and Sed^GH^ mice (Supplementary Figure S3A), the maternal preweaning environment resulted in Run^GH^ vs. Sed^GH^ DEGs in the three layers of pyramidal neurons (Fig. 3A top, Supplementary Table S3) and that they had a substantial (> 50%) overlap (Fig. 3B). Then, we found that the combined set of 87 pyramidal neuron DEGs (Fig. 3C) were again enriched in biological processes related to synapse and neurite development and function (B-H p < 0.05), such as endocytosis (of synaptic vesicles), formation of plasma membrane (e.g., neurites), and assembly of cell junctions (e.g., synapse) (Fig. 3D). Of note, although pyramidal DEGs in *dataset 1* and *2* were enriched in the same overall biological categories (e.g., synapse and neurite/neuron development), the more specific functional subcategories were somewhat different probably due, at least partly, to the difference in the animal’s prior exposure to behavioral testing.

**Figure 4:**
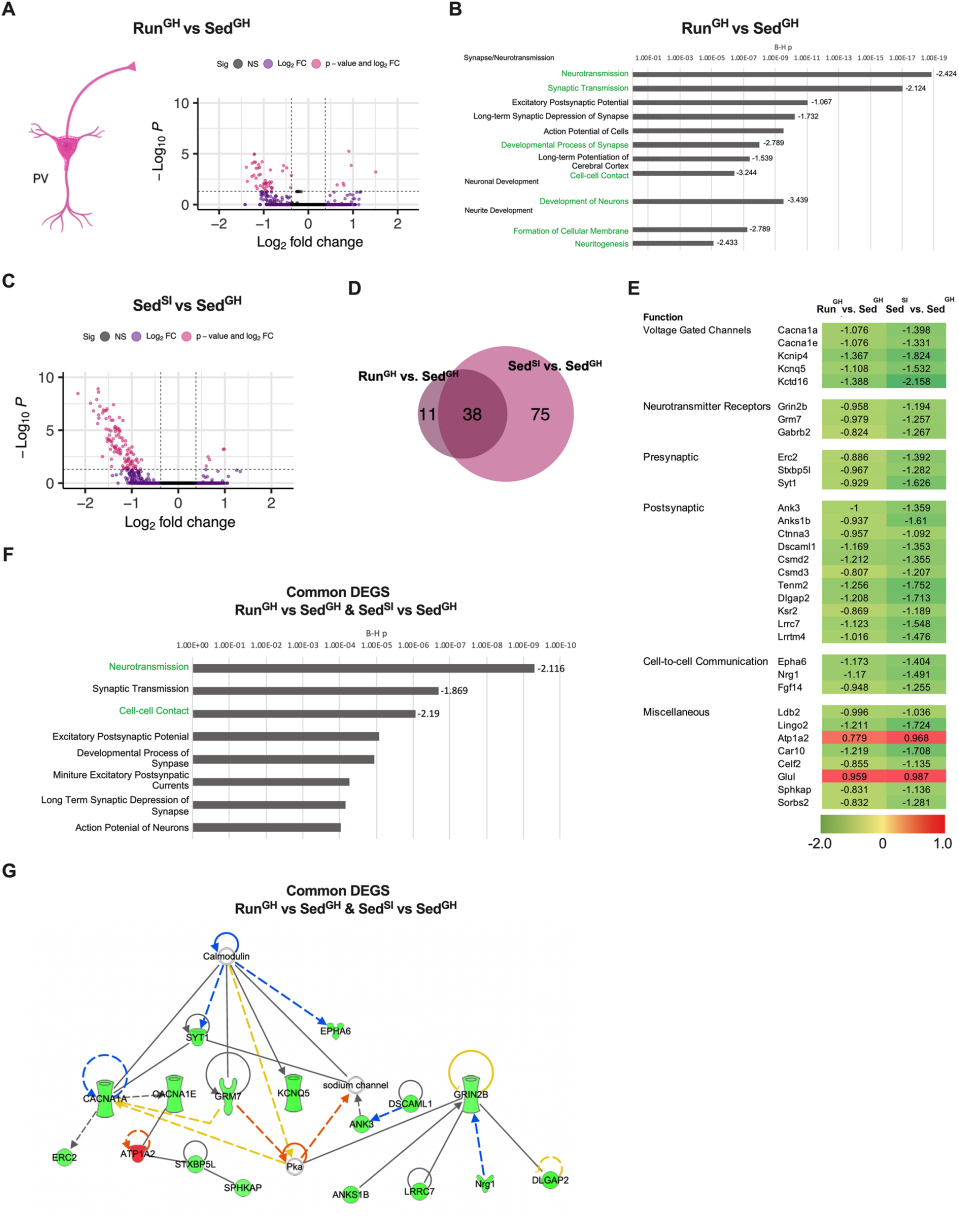
Dominance in both group-housed Run and socially isolated Sed mice is associated with the downregulation of genes encoding channels, receptors, pre/postsynaptic proteins, and adhesion molecules in PV interneurons. Data are from the four-group snRNA-Seq experiment (cohort 2-naïve, *dataset 2*). (**A**) Volcano plot representation of gene expression differences caused by the maternal Run environment in PV interneurons in the PL. (**B**) IPA biological processes enriched in Run^GH^ vs. Sed^GH^ PV interneuron DEGs. Biological processes with a significant z score (< -2) are highlighted in green. (**C**). Volcano plot representation of gene expression differences caused by social isolation in PV interneurons in the PL. (**D**). Most Run^GH^ vs. Sed^GH^ DEGs are also Sed^SI^ vs. Sed^GH^ DEGs. (**E, F**). Overlapping genes in “D” exhibit the same directionality of change and form an interactive gene network, consisting of neuronal channel, receptor, pre/postsynaptic protein and adhesion genes that together regulates synaptic communication. Green to red scale in E indicates the extent of down- and upregulation in Run^GH^ and Sed^SI^, relative to Sed^GH^. (**G**) Many of the common genes between Run^GH^ vs Sed^GH^ and Sed^SI^ vs Sed^GH^, displayed in “E”, can be networked based on known gene–gene and protein–protein interactions (IPA). The network consists of genes encoding synaptic channels, receptors, pre/postsynaptic proteins, and adhesion molecules and they together define synaptic transmission, and cell-to-cell communication in general. Since most genes are downregulated (green), the overall effect is predicted to lead to impaired network function, e.g., synaptic transmission.

Analysis of the PV interneuron expression data (*dataset 2*) revealed 49 Run^GH^ versus Sed^GH^ DEGs that were dominated by downregulated (43 of 49) genes (Fig. 3A top, Fig. 4A, Supplementary Table 3), even more so than in *dataset 1* (Supplementary Fig. 4A), suggesting their coregulation (i.e., downregulation) in Run^GH^ PL. Further, PV interneuron DEGs, relative to pyramidal neuron DEGs, were again enriched at a higher level in biological processes (B-H *p* value up to 1E-19, Fig. 4B), indicating that most DEGs and/or their encoded proteins are part of a highly interconnected gene network. The identified biological processes, like in *dataset 1* (Supplementary Fig. 4B), were related to synapse, neurite, and neuron development and many of them were associated with a significant negative z score (z < − 2) suggesting that PV interneurons in Run^GH^ PL may functionally be impaired (Fig. 4B). We note that although the pyramidal neuron and PV interneuron specific sets of Run^GH^ versus Sed^GH^ DEGs were both enriched in synapse and neurite related biological processes, the genes themselves were largely different (Supplementary Fig. 4C) indicating that the transcriptional response of pyramidal neurons and PV interneurons to the preweaning maternal environment is different. Finally, the 74 SST interneuron-specific DEGs (Fig. 3A, top) were moderately enriched in the process of endocytosis of synaptic vesicles (B-H *p* < 0.005) and the 98 astrocyte-specific DEGs (Fig. 3A, top) in the similar process of synaptic vesicle transport (B-H *p* < 0.05).

Overall, analysis of both snRNA-Seq datasets indicate that the Run maternal environment primarily affects gene expression in pyramidal neurons and PV interneurons in the PL.

### Dominance in both group-housed run and socially isolated sed mice is associated with the downregulation of genes encoding synaptic channels, receptors, and adhesion molecules in PV interneurons

A similar snRNA-seq gene expression analysis was performed with PL cells from cohort 2-naïve Sed mice that underwent social isolation (Sed^SI^; *dataset 2*), a postweaning experience that also resulted in dominance in the tube test over the Sed^GH^ mice. Social isolation induced a limited transcriptional response in layer 2/3, 5, and 6 pyramidal neurons (Fig. 3A, middle panel). However, social isolation resulted in substantial differential expression in PV interneurons (e.g., Sed^SI^ vs. Sed^GH^ DEGs) dominated by downregulation (Fig. 3A middle, Fig. 4C, Supplementary Table S4), similar to the PV interneuron-specific changes caused by the preweaning Run maternal environment (Run^GH^ vs. Sed^GH^) (Fig. 4A). Further, PV interneuron-specific Sed^SI^ vs. Sed^GH^ and Run^GH^ vs. Sed^GH^ DEGs overlapped to a significant extent (Fig. 4D), specifying a common set of 38 (33 IPA annotated) genes (Fig. 4E). All but two of these genes were downregulated by both the preweaning Run maternal and postweaning social environments and encoded voltage gated channels (Ca^++^ and K^+^), neurotransmitter receptors (NMDAR, mGluR, GABAA subunits), and presynaptic and postsynaptic proteins (Fig. 4E). Since these proteins form a neuron-to-neuron communication network (Fig. 4F, G), the downregulation of their encoding genes by the preweaning Run maternal and postweaning social environments suggests impaired inhibition of pyramidal neurons by PV interneurons in the behaviorally dominant offspring (i.e., the Sed^SI^ and Run^GH^ mice as compared to the Sed^GH^ mice). Finally, though social isolation increased dominance in Sed mice (Sed^SI^ vs. Sed^GH^), social isolation of Run mice caused a loss of dominance (Run^SI^ vs. Run^GH^) and no substantial gene expression changes in PV interneurons or in other cell types in the PL (Run^SI^ vs. Run^GH^ DEGs; Fig. 3A bottom, Supplementary Table 5). This indicates that loss of dominance in Run mice may be unrelated to gene expression changes in the PL.

**Figure 5:**
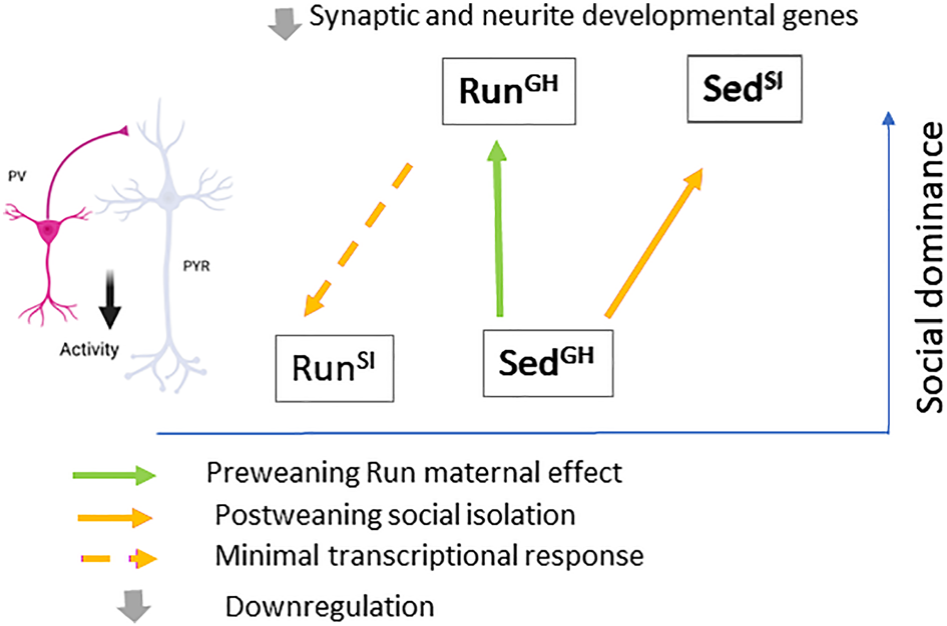
Summary of gene expression changes in PV interneurons associated with the dominance of Run^GH^ and Sed^SI^ mice and predicted reduction in PV interneuron activity in the PL microcircuit.

Taken together, gene expression profiling of different PL neurons indicates that tube test dominance, whether caused by the preweaning Run maternal environment or postweaning social isolation, is associated with the downregulation of a core set of genes in PV interneurons (Fig. 5). These genes form a highly interconnected network consisting of neuronal channel, receptor, pre/postsynaptic protein, and adhesion genes that together may regulate synaptic communication. Coordinated downregulation of these genes predicts reduced inhibition of PV interneurons on their target pyramidal neurons in the PL. This interpretation is consistent with the role of PV interneurons in disinhibiting mPFC pyramidal neurons in dominant individuals during tube test competition^30^.

## Discussion

We studied the effect of two environmental conditions on male dominance behavior in the tube test, one associated with early postnatal life (from P2 to P21/weaning) and the other with the juvenile and adolescent period of development (from weaning to adulthood). The preweaning environmental influence was represented by the mother’s physical activity, while the postweaning environment was social isolation (Fig. 5). The main finding of our work is that the dominance formed as a result of both preweaning maternal environment and postweaning social isolation (i.e., in Run^GH^ and Sed^SI^ compared to Sed^GH^) is associated with an altered transcriptional profile of PV interneurons in the PL region of the mPFC. Specifically, a set of genes involved in neurotransmission and in cell-to-cell communication in general and which include channel, receptor, pre/postsynaptic protein, and adhesion genes, were coordinately downregulated in both models suggesting impaired inhibition in the mPFC microcircuit.

The mPFC undergoes substantial development from birth until early adulthood when the mature mPFC microcircuit is established^39^. Both the preweaning suckling and postweaning juvenile/adolescent periods are critical developmental windows that are highly sensitive to environmental influences. The preweaning period is dominated by the maturation of pyramidal neurons characterized by robust synaptogenesis, synaptic pruning, and refinement of synapses^40^. Consistent with these data, the preweaning Run maternal environment resulted in persistent expression changes in genes related to synapse development and function in pyramidal neurons (Fig. 3D). Although postweaning social isolation led to the same dominance behavior as the maternal effect, it had no obvious effect on gene expression in pyramidal neurons, presumably because the highly sensitive period of pyramidal neuron synaptogenesis was closed by the time of weaning when social isolation began (Fig. 3A, middle and bottom panels). This suggests that pyramidal neurons may not have a direct role in the tube test dominance of socially isolated Sed mice.

The postweaning juvenile/adolescence period in mice is characterized by the development of inhibitory neurotransmission in the mPFC^41,42^. PV interneurons, due to their direct connectivity to pyramidal neurons and fast spiking, are important in establishing the excitatory/inhibitory balance in the adult mPFC. Consistent with the maturation of inhibitory neurons during adolescence, we found robust gene expression changes in Sed mice that were socially isolated from weaning (Fig. 3A, middle panel, Fig. 4C).

Surprisingly, the preweaning Run maternal environment resulted in similar gene expression changes in PV interneurons, (Fig. 4A, B, D) even though postweaning Run^GH^ offspring were raised in standard group-housing condition. Both the maternal environment and social isolation resulted in the coordinated downregulation of a set of PV interneuron genes related to the biological process of neurotransmission, including channel, receptor, pre/postsynaptic protein, and adhesion genes (Fig. 4E–G), implying that PV interneuron activity is likely impaired, though direct demonstration of reduced PV activity will require further experiments.

How can two environmental effects that are different in nature and developmental timing lead to similar gene expression changes, predominantly downregulation, in PV interneurons? Because PV interneurons receive long-range inputs from the thalamus and hippocampus^43^, two brain regions that process sensory information, we propose that social isolation during the juvenile and adolescent periods, due to sensory deprivation, disrupts the activity-dependent development and maturation of PV interneurons, resulting in reduced PV interneuron activity. Indeed, social isolation between P21 and P35 has been reported to cause reduced excitability and decreased activation of PV interneurons in the dorsomedial PFC^43^. The earlier timing of the Run maternal effect (preweaning) raises the possibility that the gene expression changes in pyramidal neurons have a secondary effect on PV interneuron development in a delayed fashion (postweaning) in Run^GH^ mice. Indeed, PV interneurons receive excitatory input from local pyramidal cells and thus impaired maturation of pyramidal cells, suggested by their expression profile in Run^GH^ males (Fig. 3C, D), can have a secondary effect on the development of later maturing PV interneurons.

Overall, based on the very similar gene expression changes in PV interneurons induced by the preweaning Run maternal environment and postweaning social isolation, we propose that these shared transcriptome changes, via reduced inhibition in the PL microcircuit, contribute to the dominance behavior of Run^GH^ and Sed^SI^ males in the tube test as compared to Sed^GH^. Indeed, PV interneurons project onto pyramidal cells, strongly inhibiting their activity, and disinhibition of this microcircuit has been linked to the expression of dominance behavior in the tube test^13,30^. We note, that beside their similar PV transcriptional response, Run^GH^ and Sed^SI^ males exhibited differences in behavior and gene expression. Social isolation, but not the Run maternal environment, resulted in the loss of social motivation to interact with a stranger mouse in the 3-chamber test (Fig. 1E). Further, the Run maternal environment caused gene expression changes in a wide range of cell types that included pyramidal neurons and PV and SST interneurons and astrocytes, while social isolation led to significant gene expression changes only in PV interneurons (Fig. 3A). Finally, the difference between the preweaning Run maternal and postweaning social isolation models is also reflected by the way tube test dominance is attained. While social isolation led to dominance and gene expression changes in PV interneurons in Sed mice, it prevented the expression of dominance without causing substantial PV gene expression changes in Run males that were otherwise programmed by their Run mothers to achieve dominance. We propose that social isolation abrogates the development of maternally programmed dominance but does not interfere with the maternally programmed transcriptional changes. This suggests that the mechanism underlying loss of dominance in the Run^SI^ offspring when compared to Run^GH^ offspring is either downstream of or independent from the PV interneuron transcriptional changes and the proposed reduced inhibition of pyramidal neurons.

The opposite outcome of social isolation in Sed and Run males seems to be consistent with the developmental match-mismatch theory of phenotypic adaptation^44^. Sedentary maternal environment can be considered adverse and when combined with later social isolation, another adversity, resulted in dominance, a scenario that according to the theory is a “match” between early and later life environments that typically leads to increased fitness. In contrast, Run males lost their programmed dominance following social isolation (Run^SI^), a scenario of developmental “mismatch” between early and later life environments that, according to the theory, has a cost in fitness. Although the developmental match-mismatch theory is supported by observational studies with a number of species in the wild^45–47^ and in the laboratory^48^, it is still hypothetical and lacks a plausible mechanistic explanation.

The two environmental manipulations, maternal physical activity during the postpartum period and postweaning social isolation, that were used in our experiments to increase social dominance, have relevance to human. First, maternal physical activity during the postpartum period can be considered normal as mothers, after uncomplicated childbirth, quickly resume normal daily activity. Hence, one could interpret the reduced PL expression of neurotransmission related genes and the social dominance of the offspring of physically active mothers as the ecologically likely scenario. It follows that the relative upregulation of neurotransmission related genes in Sed^GH^ mice (e.g., in comparison to Run^GH^ levels) and their submissiveness can be viewed as abnormal and related to a maternal condition that prevents mothers from resuming their typical daily activity following delivery. For example, sedentary life is associated with a proinflammatory state that could lead to the gene expression and behavioral changes in Sed males. We previously reported that the milk of Sed mothers have higher levels of cytokines that, at elevated concentrations, are proinflammatory, and that supplementing Run milk with recombinant cytokines reverts the dominance of their offspring to submissiveness^20^. Other possibilities could include the effect of the maternal microbiome, although we reported no significant differences in Sed^GH^ and Run^GH^ microbiomes during postnatal development and in adulthood^20^. Still another possibility is a change in maternal care behavior as a result of running, but we found no obvious differences between Run and Sed mothers’ care of their pups^20^.

In summary, using both a preweaning and postweaning model of increased tube test dominance, we uncovered transcriptional changes in various mPFC cell types that revealed the importance of transcriptional plasticity through gene downregulation in PV interneurons in establishing the dominance of Run^GH^ and Sed^SI^ mice compared to Sed^GH^ mice. Collectively, our study helps to understand environmentally induced transcriptional plasticity in PFC and its relationship to social dominance in the tube test.

## Methods

### Animal handling

All experimental protocols were approved by the Weill Cornell Medicine Institutional Animal Care and Use Committee (IACUC 2012-0022) following guidelines from the NIH Guide for the Care and Use of Laboratory Animals. All methods were carried out in accordance with relevant guidelines and regulations. All methods are reported in accordance with ARRIVE guidelines. All mice were group-housed up to five per cage, unless noted otherwise, throughout a 12 h light/dark cycle with lights on at 6 a.m. Food and water were available ad libitum. All experiments used C57BL/6 mice from Taconic.

### Experimental design

Experimental design to generate Run and Sed offspring, born to mothers housed in cages with or without running wheels during the postpartum period, is shown in Fig. 1A. At postnatal day 21 (P21), both Run and Sed male pups were transferred to sedentary cages and raised (i) under group-housing (Run^GH^ and Sed^GH^) condition (cohorts 1), or (ii) in group housing or social isolation (Run^GH^, Sed^GH^, Run^SI^, and Sed^SI^, cohort 2) as shown in Fig. 1A. Adult, ~ 4 months old males were tested for social dominance behavior (tube test) and social motivation (3 chamber sociability test), as described below. Cohort 1 mice were used for RNA profiling, one day after the last tube test. Further, we generated an additional cohort for RNA profiling that was identical with cohort 2 but was not tested in any behavioral assay (cohort 2-naïve). Male offspring were used in all experiments because we previously showed that Run females had similar dominance scores to their Sed counterparts, indicating no effect of maternal postpartum running on female social dominance in the tube test^20^. Cohort 1, cohort 2, and cohort 2-naïve were derived from different mothers, thus were all independent from each other. The number of animals used in each experiment is reported in the figure legends.

### Maternal postpartum running-induced offspring dominance

Parents of Sed and Run offspring were received at 7 weeks of age. They were habituated for 2 weeks before breeding started. The first litter was not used, as maternal care of primiparous females can be variable. At postnatal day P2, mothers and pups were randomly divided into 2 groups. One group was housed in cages equipped with running wheels (e.g., Run mother/offspring), while the other was housed in standard cages without running wheels (Sed mother/offspring). At P21, Run and Sed pups were weaned and raised as described in the “experimental design” section. Mothers were used only once in running experiments and were sacked afterwards to avoid the effect of repeated running on offspring behavior.

### Juvenile/adolescent separation-induced dominance

P21 Run and Sed pups were generated as described in the “experimental design” section and were housed from weaning/P21 individually in standard cages (no running wheel) until testing and tissue collection at ~ 4 months of age as shown in Fig. 1A.

### Behavioral tests

All tests were done during the light cycle (6 am–6 pm) between 12 pm and 5 pm. Sample size in all behavioral experiments was based on the number of litters/mothers. We randomly selected 1–2 male offspring from each mother. If 2 males were tested, their average behavioral score was used.

Tube test dominance: Test was performed as described previously^20^ with cohorts 1 and 2, as shown in Fig. 1A. Cohort 1 consisted of two male groups, Sed and Run, with individuals from one group competing against individuals from the other group in pairs. N was represented by the mothers, such that only one pair was created with offspring from the same two mothers. Cohort 2 consisted of four groups with individuals assigned to pairs across the groups. Again, only one pair was formed with offspring from the same two mothers. To limit the effect of winning history on dominance^11^, each male competed with only 2–3 males from the other group(s). Once mice were assigned to pairs, they were trained for three days prior to testing by placing them into one end of the tube (with divider in the middle of the tube removed) and allowing them to walk through the tube. This was repeated by putting the mouse on the other end of the tube. On day 4 (testing), a pair of trained mice were placed on the two ends of the divided tube. Once mice were fully settled, the divider was removed, and the trial started. Each pairwise competition was performed twice by switching competitors between the sides of the tube to eliminate possible side bias. The first mouse to have a front paw touch the surface of the table was declared the loser. Percent of wins for each individual was calculated.

Social rank: Right after the dominance tests between Run and Sed males, cohort 1 mice were tested for the linearity of hierarchy within individual cages by using a round robin design in which every mouse in the cage competed directly with the others in tube tests. The average number of wins determined the rank in the hierarchy within the cage. Mice with the same number of wins indicated non-linear hierarchy. Groups of cage mates were scored to have either linear (1) or non-linear (0) hierarchy.

Affiliative behavior/Social motivation: This assay was based on the 3-chamber sociability test^33^, with some modifications. Sociability was tested with cohort 2 mice two to three days after tube test. Affiliative behavior, unlike the tube test that used select individuals in pairs, could be assessed with all available males. This increased the overall number of cohort 2 animals per group in this test. Only one individual per litter was used. The test was conducted in three 10-min phases in an open plexiglass arena (50 cm × 50 cm × 34.5 cm) that mice could freely explore during each session. For the first phase, the arena was clear of objects. For the second phase, two of the same empty wire mesh cups were placed on opposite ends of the arena (diameter 4″, height 4″). For the last phase, a novel and sex-matched mouse was placed under one of the wire mesh cups. The placement of the cups and mouse was counterbalanced. Time in the interaction zone around the empty and occupied cups was measured. Interaction zone was defined as a 5 cm radius around the center of the cups. Three Run^SI^ and one Run^GH^ males spent most of the time to climb onto the occupied cup or tried to jump out from the enclosure and were excluded. Panlab SMART 3.0 video tracking software was used to track and analyze behavior.

### Isolation of prelimbic cortex (PL)

N = 3 non-brother males were processed per group. Following rapid decapitation, coronal slices were cut with a 1 mm brain block from Kent Scientific and PL was extracted. Biological replicates from each group were then pooled together. The Minute Single Nucleus Isolation Kit for Neuronal Tissue from Invent Biogen was used to isolate nuclei utilizing provided protocol. Visual inspection with a hemocytometer was used for nucleus counting.

### SnRNA-Seq from PL

First, we performed an snRNA-Seq experiment from cohort 1 Run^GH^ and Sed^GH^ mice with PL nuclei isolated one day after the last tube test (at ~ 4 months of age), generating *dataset 1.* Second, a similar snRNA-Seq experiment was performed with ~ 4 months old Run^GH^, Sed^GH^, Run^SI^, and Sed^SI^, mice that however had no prior exposure to the tube or other behavioral tests (cohort 2-naive), generating *dataset 2*). Preparation for libraries and sequencing was done by the Epigenomics Core at Weill Cornell Medicine using the Chromium Single Cell 3’ Reagent V3 Kit and sequencing via NovaSeq6000 to a depth of approximately 25,000–30,000 mean reads per cell. FASTQ files were processed using the CellRanger v6.0.0 pipeline and aligned to the mm10 transcriptome with introns included in the reference. Gene expression matrices of all samples were loaded into R and preprocessed separately using Seurat^37,49^. Nuclei with fewer than 200 genes and genes expressed in fewer than 10 nuclei were filtered out. Matrices were merged and then nuclei with fewer than 500 genes or greater than 4,000 genes and nuclei with greater than 1% mitochondrial gene reads were removed. The cutoff of greater than 4000 genes was chosen based on visualization of the number of genes per nuclei, as nuclei with a much higher than average number of genes are more likely doublets instead of single nuclei. The object was normalized using SCTransform with mitochondrial genes regressed out. Following PCA, clustering (using 35 PCs and resolution = 0.6) was performed with FindClusters. Data was then normalized using NormalizeData in the “RNA” assay. Cluster markers were identified using FindAllMarkers. For differential gene expression, clusters were combined by cell type (to a total of 9 cell types, Fig. 2C) and the animal groups were compared for each cell type individually using FindMarkers with a minimum log2FC of 1.3 and adjusted *p*-value < 0.05 for each cell type (Wilcoxon Rank Sum test, Bonferroni adjustment for the number of genes).

### Statistical analysis

The number of animals per group and the statistics used are described in the figure legend of each behavioral experiment. Data from behavioral experiments are presented as mean ± SEM. GraphPad Prism version 9.0 was used for plotting and statistical analysis (GraphPad Software), followed by the recommended post-hoc analysis. Unpaired t test was used to compare the tube test dominance of two independent groups. Two-way ANOVA with Tukey’s test for multiple comparisons was used to compare the tube test dominance of for independent groups. Mann–Whitney test was used to determine the hierarchical structure within Run and Sed cagemates. Multiple paired t tests with Sidak’s multiple comparison was used to analyze sociability. A priori level of significance at 95% confidence level was considered at *p* < 0.05.

### Supplementary Information

Supplementary Figures.

Supplementary Table S1.

Supplementary Table S2.

Supplementary Table S3.

Supplementary Table S4.

Supplementary Table S5.

## Data Availability

The data reported in this study can be found in https://www.ncbi.nlm.nih.gov/geo/query/acc.cgi?acc=GSE234726 under accession numbers GEO: GSE234726; Token for access: kbgtogcepjgjpkx. All publicly available software referenced in the text have been outlined in Materials and Methods. This paper does not report original code.
