# Supplementary material for: Single cell transcriptomic representation of social dominance in prefrontal cortex and the influence of preweaning maternal and postweaning social environment
Source: Sci Rep. 2024 Jan 25;14:2206. doi: 10.1038/s41598-024-52200-6 (PMC10810822; doi:10.1038/s41598-024-52200-6)
Supplement: Supplementary file 1 — Supplementary Figures. [file 41598_2024_52200_MOESM1_ESM.pdf]

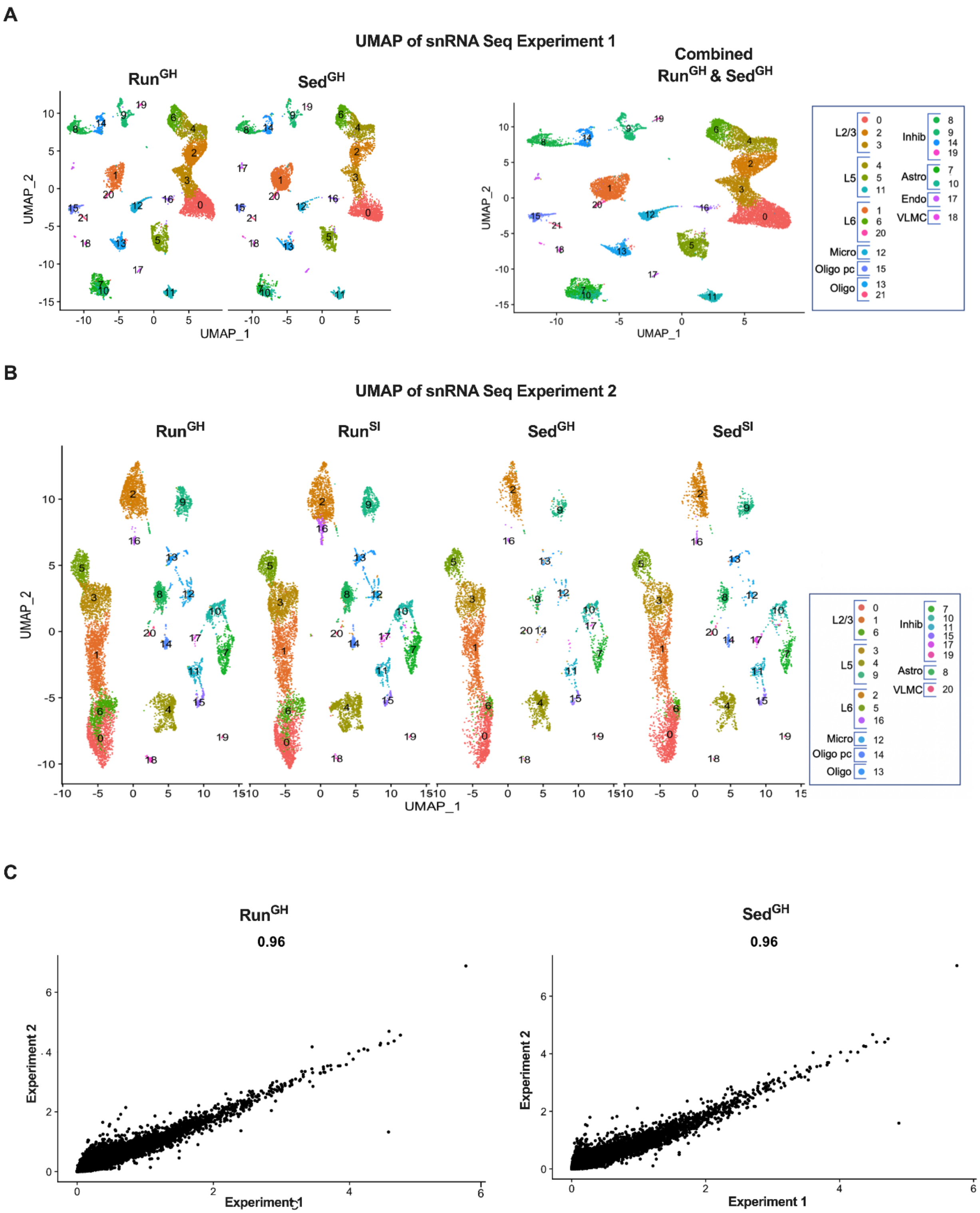

Supplementary Figure S1. Transcriptional profiles of PL single nuclei from two independent experiments/datasets. **A**. Individual and combined UMAPs for cohort 1 Run^{GH} and Sed^{GH} PL for snRNA experiment 1/dataset 1, showing all identified neuronal and nonneuronal cell types. L=cortical layer, Micro=microglia, Oligo pc=oligodendrocyte precursor, Inhib=inhibitory neurons, Endo=endothelial cell, VLMC= vascular and leptomeningeal cells. **B**. Individual UMAPs for cohort 2-naïve Run^{GH}, Sed^{GH}, Run^{SI}, and Sed^{SI} PL for snRNA experiment 2/dataset 2, See combined UMAP in Figure 2C. **C**. Correlation of total gene expression of PL between snRNA-Seq datasets 1 and 2. X and Y coordinates: relative expression levels.

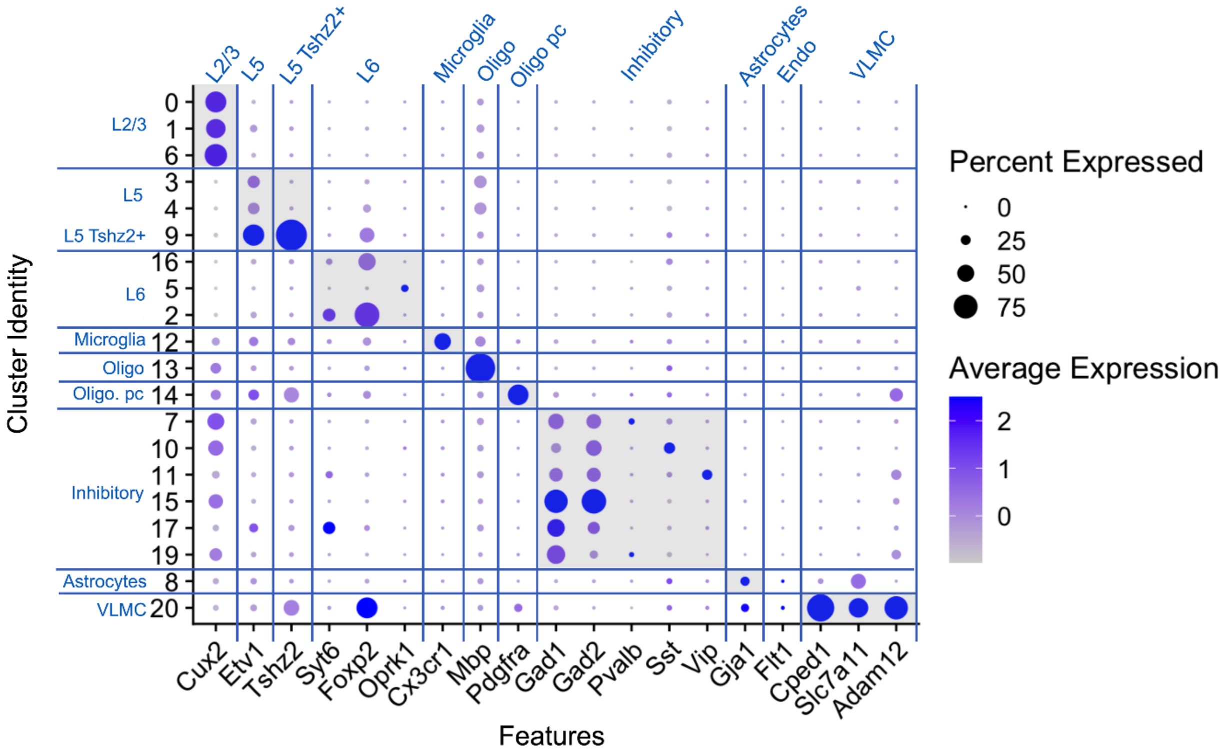

Supplementary Figure S2. Average expression of canonical marker genes for each cluster in a combined set of cohort 2-naive Run^{GH}, Sed^{GH}, Run^{SI} and Sed^{SI} PL. Genes listed on the x-axis and clusters and their associated cell type on the y-axis.

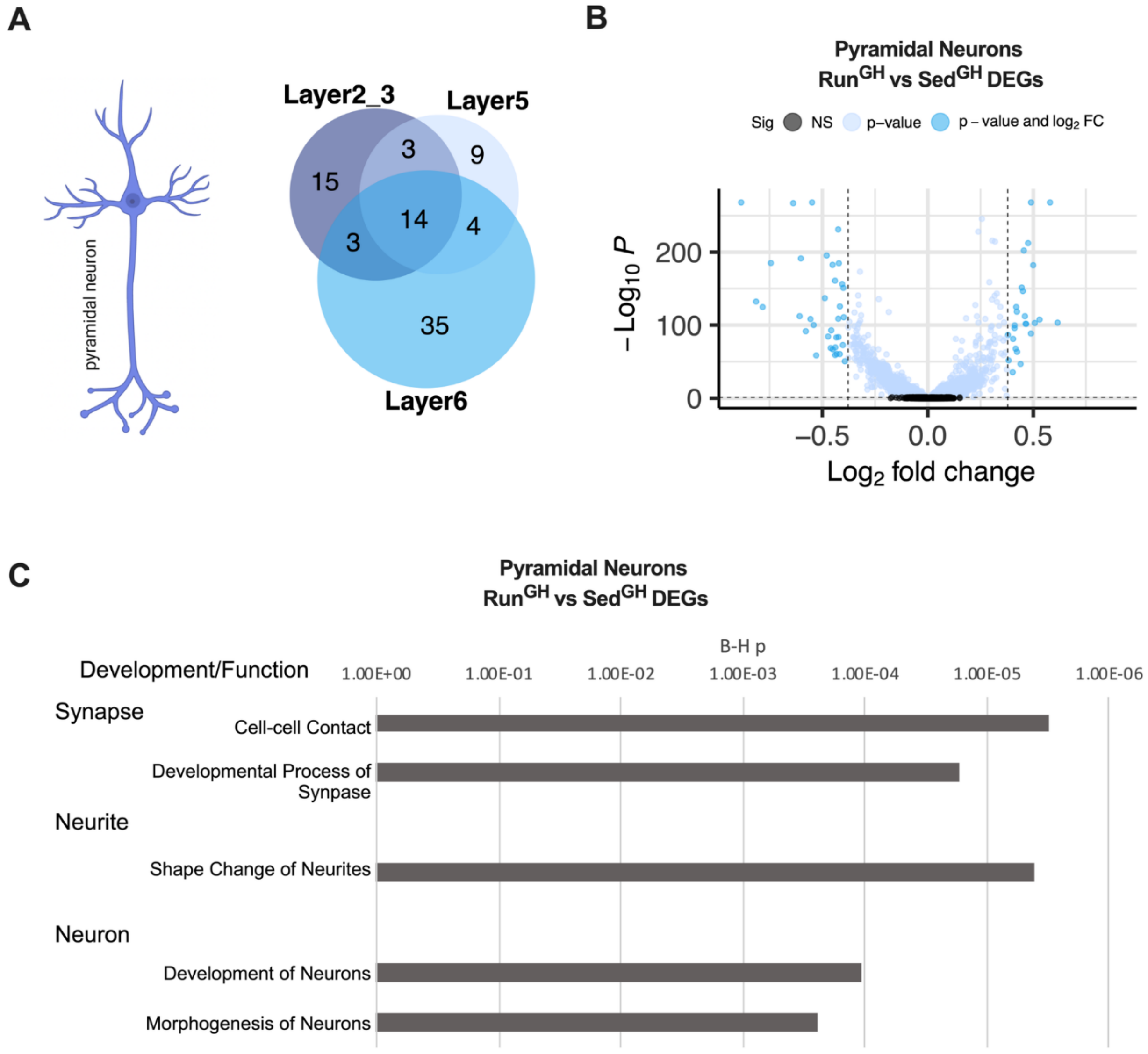

Supplementary Figure S3. Analysis of pyramidal neuron-specific Run^{GH} vs. Sed^{GH} DEGs from snRNA-Seq, dataset 1 (cohort 1). **A**. DEGs corresponding to the three layers of PL highly overlap. **B**. Volcano plot representation of gene expression differences caused by the maternal Run environment in pyramidal neurons in the PL. **C**. IPA biological processes enriched in Run^{GH} vs. Sed^{GH} pyramidal neuron DEGs.

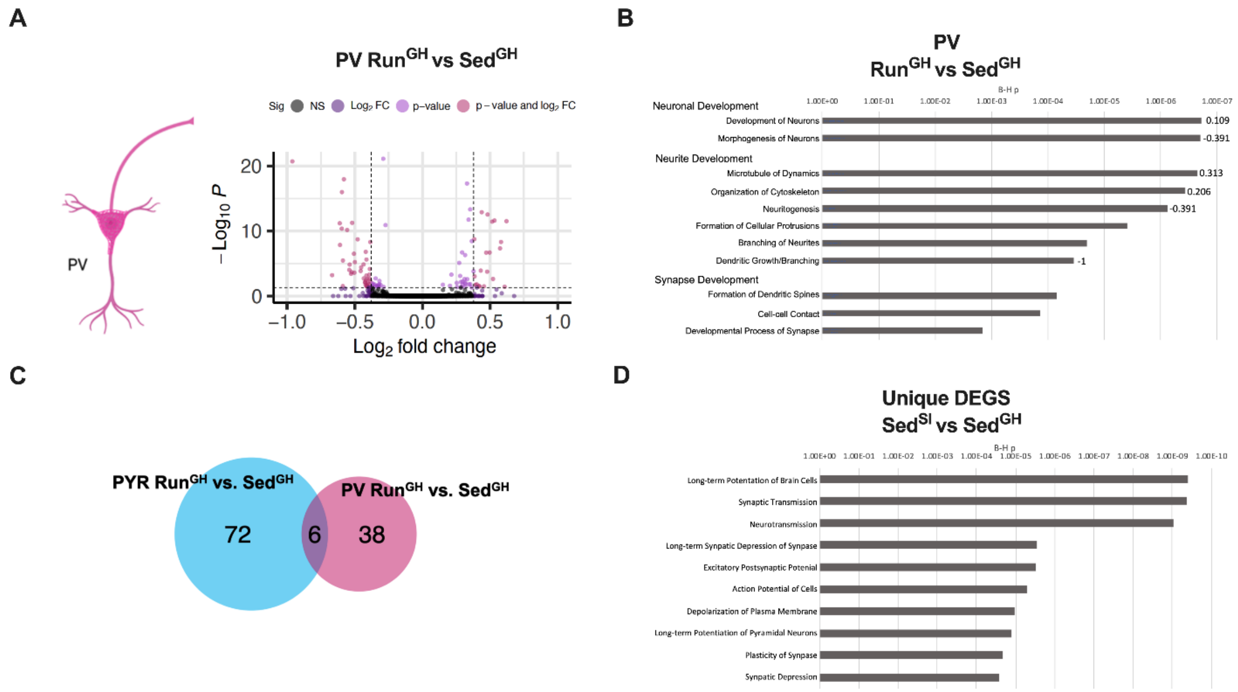

Supplementary Figure S4. Analysis of PV interneuron-specific Run^{GH} vs. Sed^{GH} DEGs from snRNA-Seq, from dataset 1 (cohort 1). **A**. Volcano plot representation of gene expression differences caused by the maternal Run environment in PV interneurons in the PL. **B**. IPA biological processes enriched in Run^{GH} vs. Sed^{GH} PV interneuron DEGs. Z scores are indicated on top of the columns. **C**. Minimal overlap between pyramidal neuron and PV interneuron Run^{GH} vs. Sed^{GH} DEGs. **D**. IPA biological processes enriched in unique Sed^{SI} vs Sed^{GH} DEGs (e.g., that are not shared with Run^{GH} vs Sed^{GH} DEGs).
